# Model-Based Estimation of Colorectal Cancer Screening and Outcomes During the COVID-19 Pandemic

**DOI:** 10.1001/jamanetworkopen.2021.6454

**Published:** 2021-04-12

**Authors:** Rachel B. Issaka, Preston Taylor, Anand Baxi, John M. Inadomi, Scott D. Ramsey, Joshua Roth

**Affiliations:** 1 Clinical Research Division, Fred Hutchinson Cancer Research Center, Seattle, Washington; 2 Hutchinson Institute for Cancer Outcomes Research, Fred Hutchinson Cancer Research Center, Seattle, Washington; 3 Division of Gastroenterology, University of Washington School of Medicine, Seattle; 4 Department of Medicine, University of Utah School of Medicine, Salt Lake City

## Abstract

**Importance:**

COVID-19 has decreased colorectal cancer screenings.

**Objective:**

To estimate the degree to which expanding fecal immunochemical test–based colorectal cancer screening participation during the COVID-19 pandemic is associated with clinical outcomes.

**Design, Setting, and Participants:**

A previously developed simulation model was adopted to estimate how much COVID-19 may have contributed to colorectal cancer outcomes. The model included the US population estimated to have completed colorectal cancer screening pre–COVID-19 according the American Cancer Society. The model was designed to estimate colorectal cancer outcomes between 2020 and 2023. This analysis was completed between July and December 2020.

**Exposures:**

Adults screened for colorectal cancer and colorectal cancer cases detected by stage.

**Main Outcomes and Measures:**

Estimates of colorectal cancer outcomes across 4 scenarios: (1) 9 months of 50% colorectal cancer screenings followed by 21 months of 75% colorectal cancer screenings; (2) 18 months of 50% screening followed by 12 months of 75% screening; (3) scenario 1 with increased use of fecal immunochemical tests; and (4) scenario 2 with increased use of fecal immunochemical tests.

**Results:**

In our simulation model, COVID-19–related reductions in care utilization resulted in an estimated 1 176 942 to 2 014 164 fewer colorectal cancer screenings, 8346 to 12 894 fewer colorectal cancer diagnoses, and 6113 to 9301 fewer early-stage colorectal cancer diagnoses between 2020 and 2023. With an abbreviated period of reduced colorectal cancer screenings, increasing fecal immunochemical test use was associated with an estimated additional 588 844 colorectal cancer screenings and 2836 colorectal cancer diagnoses, of which 1953 (68.9%) were early stage. In the event of a prolonged period of reduced colorectal cancer screenings, increasing fecal immunochemical test use was associated with an estimated additional 655 825 colorectal cancer screenings and 2715 colorectal cancer diagnoses, of which 1944 (71.6%) were early stage.

**Conclusions and Relevance:**

These results suggest that the increased use of fecal immunochemical tests during the COVID-19 pandemic was associated with increased colorectal cancer screening participation and more colorectal cancer diagnoses at earlier stages. If our estimates are borne out in real-world clinical practice, increasing fecal immunochemical test–based colorectal cancer screening participation during the COVID-19 pandemic could mitigate the consequences of reduced screening rates during the pandemic for colorectal cancer outcomes.

## Key Points

**Question:**

What 3-year clinical outcomes are associated with expanding fecal immunochemical test (FIT)–based colorectal cancer screening participation during the COVID-19 pandemic?

**Findings:**

In this modeling study, increasing FIT use from 15% to 22% over a 3-year period to offset COVID-19–related declines in colonoscopy screening was associated with an additional 655 825 colorectal cancer screenings and 2715 colorectal cancer diagnoses, of which 72% were early stage.

**Meaning:**

These findings suggest that increasing FIT use for colorectal cancer screening during the COVID-19 pandemic may mitigate the consequences of reduced screening rates caused by the pandemic for colorectal cancer outcomes.

## Teaser

This modeling study estimates COVID-19–related changes in rates of colorectal cancer screenings and associated outcomes and estimates the degree to which expanded fecal immunochemical testing could potentially mitigate these outcomes.

## Introduction

Colorectal cancer (CRC) is the second-leading cause of cancer death in the US.^1^ Despite clear evidence that screening by colonoscopy and stool-based tests is cost-effective^2^ and saves lives,^3,4^ screening remains underutilized.^5^ In the US, only 67% of adults between the ages of 50 and 75 are up to date with CRC screening,^6^ and in Federally Qualified Health Centers, the largest providers of care to underinsured and uninsured individuals, only 44% of the population is up to date.^7^ Both estimates fall short of the National Colorectal Roundtable goal of achieving 80% adherence to CRC screening.^8^

In response to the COVID-19 pandemic, the Centers for Medicare & Medicaid Services (CMS) initially recommended that all nonurgent surgical and medical procedures, including screening colonoscopies, be delayed.^9^ This recommendation resulted in a 90% decline in CRC screenings, 32% decline in new CRC diagnoses, and 53% decline in CRC surgical procedures by mid-April 2020 compared with a year prior.^10^ At the time of our analysis, screening colonoscopies remained approximately 50% lower than when the pandemic began.^11^

COVID-19 delays in CRC screening will lead to delays in diagnoses, stage progression for individuals with undiagnosed cancer, and increase CRC mortality. Many countries have implemented population-based CRC screening with the fecal immunochemical test (FIT) as the dominant screening method. While colonoscopy is the most commonly used CRC screening test in the US,^12^ there is increased uptake of stool-based screening tests in large integrated health systems^13^ and in resource-constrained settings where patients may have a preference for noninvasive screening modalities.^14^ In light of the decline in CRC screenings, especially colonoscopy-based screenings, increased use of FIT during the pandemic could potentially limit the deleterious public health consequences of COVID-19 on CRC mortality. FIT is an inexpensive, annual, at-home CRC screening method that checks for blood in stool and can be distributed and returned by mail.^15,16^ A meta-analysis that examined test characteristics found FIT had a pooled sensitivity of 79%, specificity of 94%, and overall diagnostic accuracy of 95% for CRC.^17^

The objective of this study was to estimate how expanding FIT-based CRC screening would affect screening participation during the COVID-19 pandemic. We compared 4 COVID-19 scenarios, during which CRC screening was reduced for variable amounts of time with or without increased used of FIT-based screening. Our findings are designed to assist medical professionals as they assess clinical tradeoffs of approaches to increase CRC screening participation in the US during the COVID-19 pandemic and beyond.

## Methods

### Overview

**Figure 1.**
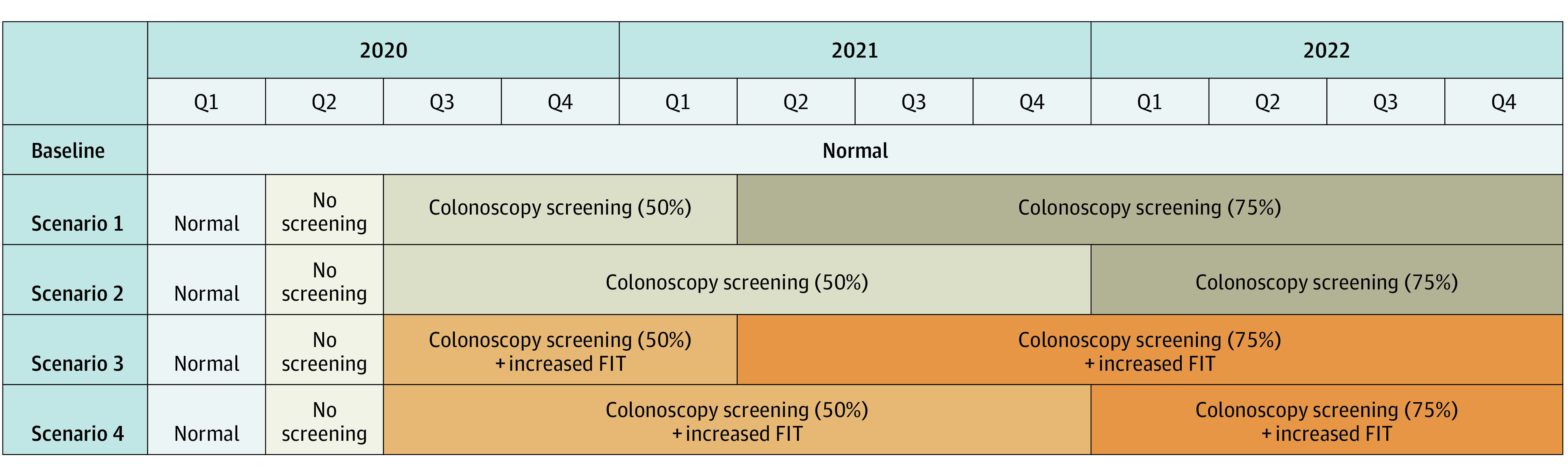
Schematic of Modeled COVID-19 Scenarios

We adopted a previously developed simulation model^18^ in Excel version 16.39 (Microsoft) to estimate the 3-year CRC outcomes for average-risk individuals eligible for CRC screening according to the US Preventive Services Task Force.^19^ We focused on a short-term (3-year) time horizon to align with the decision-making needs of many health systems and policy makers in the US.^20^ Our approach synthesized evidence from validation studies from colonoscopy^21^ and FIT-based CRC screening tests^17,22,23,24^ and screening statistics from the American Cancer Society (ACS).^6^ Our model assumed colonoscopy sensitivity for CRC of 95% (95% CI, 90%-100%)^25^ and CRC prevalence of 0.08% (95% CI, 0.07%-0.09%) based on an average of 3 published studies.^22,26,27^ We assumed sensitivity of FIT for CRC of 79% (95% CI, 69%-85%),^17^ a FIT positivity rate of 7% (95% CI, 6.3%-7.7%),^22^ and that 65% of those with an abnormal FIT result would complete a diagnostic colonoscopy.^38,39^ A simplified schematic of the model structure is shown in Figure 1, and Table 1 provides the model input values, uncertainty ranges, and data sources used in the study.

**Table 1.** Model Input Values and Data Sources

Input	Baseline case value, % (95% CI)^a^	Source
Screening test characteristics: colorectal cancer		
Colonoscopy, sensitivity	95.0 (90.0-100)	Vijan,^25^ 2001
Colonoscopy, CRC prevalence	0.8 (0.7-0.9)	Church,^26^ 2014; Imperiale,^22^ 2014; Ferlitsch,^27^ 2011
FIT, sensitivity	79 (69-85)	Lee,^17^ 2014
FIT, classified positive	7 (6.3-7.7)	Imperiale,^22^ 2014
Diagnostic follow-up inputs		
FIT, proportion adherent to diagnostic colonoscopy	65.0 (52.0-78.0)	Chubak,^38^ 2016; Issaka, ^39^ 2017
Stage distribution: screen-detected with FIT		
Stage I proportion	0.367	Roth,^18^ 2019
Stage II proportion	0.347	Roth,^18^ 2019
Stage III proportion	0.217	Roth,^18^ 2019
Stage IV proportion	0.069	Roth,^18^ 2019
Stage distribution: screen detected colonoscopy		
Stage I proportion	0.34	Knudsen,^21^ 2016
Stage II proportion	0.36	Knudsen,^21^ 2016
Stage III proportion	0.19	Knudsen,^21^ 2016
Stage IV proportion	0.11	Knudsen,^21^ 2016
Stage distribution: clinically detected colonoscopy		
Stage I proportion	0.18	Knudsen,^21^ 2016
Stage II proportion	0.34	Knudsen,^21^ 2016
Stage III proportion	0.23	Knudsen,^21^ 2016
Stage IV proportion	0.25	Knudsen,^21^ 2016

Abbreviation: FIT, fecal immunochemical test.

^a^ Results rounded to the first decimal place, except when not provided by the source study.

The decision modeling approach used in this analysis follows the methods of budget impact modeling as presented in the International Society for Pharmacoeconomics and Outcomes Research guidelines for good practice in reporting budget impact and clinical outcomes findings,^20^ but we focus only on clinical outcomes and not on health plan expenditure. This approach was taken because several analyses have confirmed that CRC screening by stool-based tests and colonoscopy are cost-effective.^28,29^ We also adhered to the Consolidated Health Economic Evaluation Reporting Standards (CHEERS) reporting guideline for economic analyses. The cohort-level decision model used in this study derived all inputs from publicly available data and therefore the institutional review board at the Fred Hutchinson Cancer Research Center did not consider this study human subjects research that required approval. Data analysis was completed between July and December 2020.

### Population and Setting

**Figure 2.**
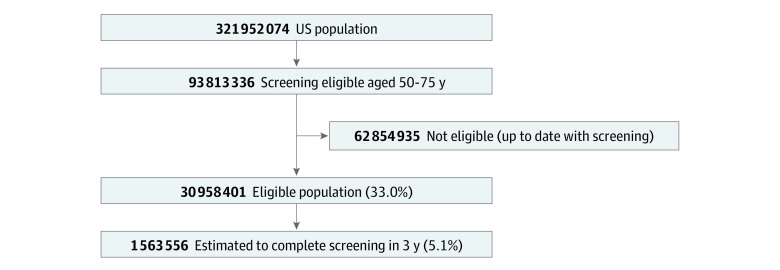
Baseline Screening Population

We first modeled the US population estimated to complete screening either by colonoscopy or FIT based on pre-COVID ACS CRC Facts and Figures.^6^ Age and gender distributions were based on the 2016 projection of the 2010 US Census. The distribution of individuals eligible for CRC screening is shown in Figure 2. Based on historical ACS data, 67% percent of eligible adults completed CRC screening, with 85% completing screening by colonoscopy and 15% by stool test. FIT was selected as our preferred stool test. Assuming screening uptake over the next 3 years (2020 to 2023) is similar to the 2017 to 2020 trends,^6^ 5.1% of eligible individuals will complete CRC screening over this time horizon.

### COVID-19 CRC Screening Scenarios

The 4 COVID-19 scenarios varied with respect to 2 dimensions: first, the COVID-19 dispersion profile (ie, whether or not COVID-19 cases led to local or national guidance to suspend elective procedures), and second, our collective response to improve CRC screening during the pandemic (whether or not there is an increase in FIT-based screening programs in response to decreased CRC screenings).

#### COVID-19 Dispersion Profile

Because of a combination of COVID-19–related enhanced patient protections, hospital reorganizations, decreased staff, patient fear, and CRC screening rates at the time of our analysis, our models assumed across modalities that CRC screenings were approximately 50% lower than prior to COVID-19. For the purpose of our model, we also assumed that the COVID-19 dispersion profile would result in varying durations of reduced CRC screenings until COVID-19 vaccines were widely available, enabling screenings to return to pre–COVID-19 volumes. Based on our estimates of individuals who would complete screening (Figure 2), we adjusted CRC screening by colonoscopy or FIT to 50% or 75% over varying time periods to match our proposed scenarios.

Anticipating that CRC screenings will be disrupted over a 3-year (36-month) period, scenario 1 assumed 3 months of normal screening (pre–COVID-19), 3 months without any CRC screening (first peak of COVID-19), and 9 months during which CRC screenings would be 50% of prepandemic volume followed by 21 months during which CRC screenings would be 75% of prepandemic volume. Scenario 2 assumed a similar initial reduction in CRC screenings, but that prolonged COVID-19 cases would lead to 18 months during which CRC screenings would be 50% of prepandemic volumes followed by 12 months during which CRC screenings would be 75% of prepandemic volumes. Scenario 3 mirrored scenario 1 while scenario 4 mirrored scenario 2, except that both scenarios 3 and 4 included increased FIT-based screenings to address reductions in CRC screenings due to COVID-19 (Figure 1).

#### FIT Use in Response to COVID-19

Compared with the baseline of 15% FIT-based CRC screenings, COVID-19 related delays resulted in 8% FIT use in scenarios 1 and 2 over the 3-year period. FIT use in scenarios 3 and 4 were modeled using the median value of FIT completion in Kaiser Permanente (22.5%),^13^ a national leader in FIT-based CRC screening, and this value was applied to both the 25% or 50% estimates of the population that would not otherwise complete CRC screening due to COVID-19. This deliberate effort to increase FIT use was assumed to result in 20% to 22% FIT-based CRC screenings in scenarios 3 and 4 over the 3-year period (Table 2).

**Table 2.** Colonoscopy and FIT Uptake Estimates by Scenario Over the 3-Year Model Period^a^

Scenario	Estimated cases, %				
	Baseline	Scenario 1	Scenario 2	Scenario 3	Scenario 4
Year 1					
Colonoscopy	85.0	42.0	42.0	42.0	42.0
FIT	15.0	8.0	8.0	17.0	17.0
Not screened	0	50.0	50.0	40.0	40.0
Year 2					
Colonoscopy	85.0	58.0	42.0	58.0	42.0
FIT	15.0	8.0	8.0	22.0	27.0
Not screened	0	34.0	50.0	19.0	31.0
Year 3					
Colonoscopy	85.0	64.0	64.0	64.0	64.0
FIT	15.0	8.0	8.0	21.0	21.0
Not screened	0	29.0	29.0	15.0	15.0
Year 1-3 average					
Colonoscopy	85.0	55.0	49.0	55.0	49.0
FIT	15.0	8.0	8.0	20.0	22.0
Not screened	0	38.0	43.0	25.0	29.0

Abbreviation: FIT, fecal immunochemical test.

^a^ Within each scenario, colorectal cancer screening changes from year 1 to year 3 based on the COVID-19 dispersion profile and uptake of colonoscopy or FIT.

#### Colonoscopy Use in Response to COVID-19

After adjusting for increased FIT use, compared with the baseline of 85% colonoscopy-based screenings, COVID-19–related delays resulted in 55% colonoscopy use in scenarios 1 and 3 and 49% colonoscopy use in scenarios 2 and 4 over the 3-year period (Table 2).

### Colorectal Cancer Stage Distribution at Diagnosis

To estimate the stage distribution of CRC cases detected for each screening method, the population prevalence estimates were multiplied by the reported stage-specific detection rates.^22,23^ The estimated distributions for screening colonoscopy were 34% (stage I), 36% (stage II), 19% (stage III), and 11% (stage IV); distributions for FIT were 36.7% (stage I), 34.7% (stage II), 21.7% (stage III), and 6.9% (stage IV).^21^ We used the Surveillance, Epidemiology, and End Results incidence rate from 1975 to 1979 to estimate the proportion of nonadherent patients and false-negative patients who were diagnosed with CRC after developing symptoms. These individuals with symptoms detected had a more advanced-stage distribution (18% for stage I, 34% for stage II, 23% for stage III, and 25% for stage IV).^21^

### Outcomes

We used the decision model framework to estimate the counts and proportions of people in the US who completed CRC screening, were newly diagnosed with CRC, or had CRC detected at an early stage (ie, stages I and II) or late stage (stages III and IV) for the alternative screening scenarios. These outcomes were calculated by applying CRC screening test characteristics (and clinical detection characteristics for those refusing screening) to the screening-eligible US population over a 3-year time horizon (representing 2020-2023). The outcomes from each scenario were compared with the baseline, referent scenario. Additionally, the outcomes from scenario 1 and 3 were compared with each other as were the outcomes from scenarios 2 and 4.

## Results

Using CRC screening uptake between 2017 and 2020 as a reference,^6^ our baseline scenario assumed 1 563 556 eligible individuals (5.1%) will complete CRC screening by colonoscopy or FIT per year over the next 3 years, totaling 4 690 668 screened individuals (Figure 2). Of the 34 323 CRC cases, 33 747 (98.3%) would be detected by screening and 576 (1.7%) would be clinically detected by symptoms. Among all the CRC cases, 23 964 (69.8%) would be detected at an early stage (Table 3).

**Table 3.** Number of People Screened for Colorectal Cancer, New CRC Cases, and Early Stage CRC Cases Detected Across Modeled COVID-19 Scenarios

	No. (% difference)				
	Baseline	Scenario 1	Scenario 2	Scenario 3	Scenario 4
People who complete CRC screening, No.	4 690 668	2 924 880	2 676 503	3 513 725	3 332 328
Scenario vs baseline	1 [Reference]	1 765 788 (−37.6)	2 014 165 (−42.9)	1 176 943 (−25.1)	1 358 340 (−29.0)
Scenario 3 vs 1	NA	1 [Reference]	NA	588 845 (+20.1)	NA
Scenario 4 vs 2	NA	NA	1 [Reference]	NA	655 825 (+24.5)
New CRC cases, No.	34 323	23 141	21 429	25 977	24 144
Screening detected CRC, No. (%)	33 747 (98.3)	21 359 (92.3)	19 436 (90.7)	19 274 (89.9)	22 180 (91.9)
Symptom detected CRC, No. (%)	576 (1.7)	1782 (7.7)	1993 (9.3)	2155 (10.1)	1964 (8.1)
Scenario vs baseline	1 [Reference]	11 182 (−32.6)	12 894 (−37.6)	8346 (−24.3)	10 179 (−29.7)
Scenario 3 vs 1	NA	1 [Reference]	NA	2836 (+12.3)	NA
Scenario 4 vs 2	NA	NA	1 [Reference]	NA	2715 (+12.7)
Early stage CRC, No. (%)	23 964 (69.8)	15 898 (68.7)	14 663 (68.4)	17 851 (68.7)	16 607 (68.8)

Abbreviations: CRC, Colorectal Cancer; NA, not applicable.

### Scenario 1: Reduced Colonoscopy-Based CRC Screening Without Prolonged COVID-19 Course

If COVID-19–related declines in colonoscopy-based CRC screenings are limited in duration (9 months), an estimated 1 765 788 (37.6%) fewer patients would complete screening and 11 182 (32.6%) fewer cancers would be diagnosed without increased use of noncolonoscopy screening tests compared with the baseline scenario. Of the 23 141 CRC cases diagnosed over the 3-year period, an estimated 21 359 (92.3%) would be detected by screening and 1782 (7.7%) would be symptom-detected. Among all the CRC cases, an estimated 15 898 (68.7%) would be detected at an early stage.

### Scenario 2: Reduced Colonoscopy-Based CRC Screening With a Prolonged COVID-19 Course

If COVID-19–related declines in colonoscopy-based CRC screenings persist (ie, 18 months), an estimated 2 014 165 (42.9%) fewer people would complete CRC screening and 12 894 (37.6%) fewer cancers would be diagnosed compared with the baseline scenario. Of the 21 429 CRC cases diagnosed over the 3-year period, an estimated 19 436 (90.7%) would be detected by screening and 1993 (9.3%) would be symptom-detected. Among all the CRC cases, an estimated 14 663 (68.4%) would be detected at an early stage.

### Scenario 3: Reduced Colonoscopy-Based CRC Screening Without a Prolonged COVID-19 Course and Increased FIT Use

If COVID-19–related declines in colonoscopy-based CRC screenings are limited in duration (ie, lasting 9 months), by increasing FIT-based screening to 20% over a 3-year period, an estimated 1 176 943 (25.1%) fewer people would complete CRC screening and 8346 (24.3%) fewer cancers would be diagnosed compared with the baseline scenario. Among all the CRC cases, an estimated 17 851 (68.7%) would be detected at an early stage. Of the 21 429 CRC cases diagnosed over the 3-year period, an estimated 19 274 (89.9%) would be detected by screening and 2155 (10.1%) would be symptom-detected. In contrast to scenario 1, increasing FIT-based screening would result in an additional 588 845 (20.1%) of eligible individuals completing CRC screening and an estimated additional 2836 (12.3%) of CRC diagnoses, of which 1953 cases (68.9%) would be detected at an early stage.

### Scenario 4: Reduced Colonoscopy-Based CRC Screening With a Prolonged COVID-19 Course and Increased FIT Use

If COVID-19–related declines in colonoscopy-based CRC screenings persist (ie, lasting 18 months), by increasing FIT-based screening to 22% over a 3-year period, an estimated 1 358 340 (29.0%) fewer people would complete CRC screening and 10 179 (29.7%) fewer cancers would be diagnosed compared with the baseline scenario. Among all the CRC cases, an estimated 16 607 (68.8%) would be detected at an early stage. Of the 24 144 CRC cases diagnosed over the 3-year period; an estimated 22 180 (91.9%) would be detected by screening and 1964 (8.1%) would be clinically detected. In contrast to scenario 2, increasing FIT-based screening would result in an additional 655 825 individuals (24.5%) completing CRC screening and an additional 2715 CRC diagnoses (12.7%), of which 1944 (71.6%) would be detected at an early stage (Table 3).

## Discussion

COVID-19 has caused an unprecedented disruption in cancer prevention, including CRC screening. The objective of this study was to estimate the clinical impact of expanding FIT-based CRC screening participation during the COVID-19 pandemic. Our analysis suggests that in an extended period of limited CRC screenings due to the pandemic, up to 43% of eligible adults could remain unscreened without increased use of FIT to offset decreased screenings, resulting in approximately 13 000 fewer CRC diagnoses and 9000 fewer early stage CRC diagnoses. However, increasing FIT use during this time could reduce the proportion of eligible adults to 29% and detect an estimated additional 2715 CRC cases, the majority of which would be detected at an early stage. The potential benefits could be even greater if FIT use increased beyond our modest assumptions.

To date, over 450 000 COVID-19–related deaths have been reported in the US. Early shelter-in-place ordinances led to an initial 90% decline in CRC screenings, and while many screening services have resumed, participation remains lower than when the pandemic began. Ongoing declines in CRC screening participation are likely due to a combination of procedure backlogs, health system reductions in services offered due to financial losses, loss of employment-associated health insurance, and patient fear. Altogether COVID-19–associated decreases in screening will lead to increased mortality from CRC and other preventable cancers,^30^ CRC is one of the few cancers for which there are multiple screening options. Through organized mailed FIT outreach, Kaiser Permanente increased CRC screening participation by 44% and reduced CRC mortality by 52% over a 5-year period.^13^ Increased use of FIT, especially through organized mailed outreach, therefore has the potential to mitigate the negative consequences of COVID-19 on CRC outcomes.^31,32^

FIT presents a widely accepted, scalable, and cost-effective screening option^29^ that can be delivered and completed at home and could minimize the public health impact on CRC outcomes from COVID-19. Our analysis reveals that even with increased use of FIT, 121 fewer CRC cases are diagnosed if COVID-19 delays in CRC screening are prolonged (scenario 4) vs abbreviated (scenario 3). This difference in CRC cases is likely because of a lack of access to screening colonoscopies, and also highlights the difference in test performance between FIT and colonoscopy. Yet, the number of additional CRC cases detected with increased FIT-based screening (2715 cases) is greater than the number of CRC cases missed (121 cases) and justifies considering increased use of FIT during the pandemic.

Several recent modeling studies have highlighted how COVID-19 delays may potentially increase cancer stage and avoidable cancer deaths nationally^30^ and globally.^33,34^ In the United Kingdom, modeling studies also suggest that stratifying individuals by FIT positivity thresholds during periods of decreased colonoscopy access could decrease preventable CRC deaths attributable to COVID-19 endoscopic delays.^35^

Our simulation model inputs included historical data about CRC incidence, stage at diagnosis, and stage shift associated with CRC screening. This type of decision model tool is not intended to provide precise estimates of future outcomes, but rather is a framework intended to inform decision makers about the likely range of changes in clinical outcomes that may result from different model assumptions. Our study extends the existing literature by modeling how the potential impact from CRC screening delays could be mitigated by increased use of FIT. Our prior studies^29,40^ have demonstrated that mailed FIT effectively increases CRC screening participation. Taken together, our findings provide practical options that practices can consider as part of their COVID-19 response to CRC screening declines.

### Limitations

This study had several limitations worth noting. First, optimally implementing FIT-based screening is critical to improving CRC mortality, but this was beyond the scope of the study. Second, as with any model-based analysis, the accuracy of our estimates is dependent on the validity of the baseline assumptions and estimates used. Our model used CRC outcomes estimates from different cohorts because there are no randomized controlled trials comparing colonoscopy with FIT, although several are now in progress.^36^ Our model also assumes that screening disruptions due to the COVID-19 pandemic will affect screening participation for a 3-year period. We believe these assumptions are sound given the course of COVID-19, the impact of the pandemic on cancer screenings in the US, and vaccine hesitancy^37^ that may delay achieving herd immunity and a return to prepandemic screening patterns. Third, our model assumed that CRC screening participation between 2020 and 2023 will mirror screening participation from 2017 to 2020 and used a modest increase in FIT use across the scenarios. However, this conservative approach means that our analysis underestimated, rather than overestimated, the potential impact of COVID-19 on CRC screening. Fourth, a critical component of any FIT-based screening program is the completion of a diagnostic colonoscopy after an abnormal result. Our model assumed that 65% of those with an abnormal result would complete a colonoscopy, an average from programs with the highest and lowest colonoscopy completion rates,^38,39^ and that colonoscopy would be available to all patients with an abnormal FIT result. However, the model estimates could differ if colonoscopy completion or availability was much lower than our estimated value. Fifth, our model did not account for the individuals who require repeat screening or those who remain unscreened for CRC despite best efforts because of pandemic-related delays and other factors.

## Conclusions

Prior to the pandemic, an estimated 33% of eligible US adults had not completed CRC screening. COVID-19 colonoscopy delays threaten to undo decades worth of progress to decrease CRC mortality through increased CRC screening and awareness. In this modeling study, we found that increased use of FIT-based screening during the COVID-19 pandemic increased CRC screening participation and resulted in more CRC diagnoses at earlier stages than no increased use of FIT. If our estimates are borne out in real-world clinical practice, proactive strategies such as increased use of FIT-based CRC screening—particularly through organized mailed outreach—may help limit the undoing of public health progress in CRC and, perhaps, even contribute to achieving the National Colorectal Roundtable goal of 80% adherence to screening nationwide.
